# Observation of Quantized and Partial Quantized Conductance in Polymer-Suspended Graphene Nanoplatelets

**DOI:** 10.1186/s11671-016-1387-8

**Published:** 2016-04-05

**Authors:** Yuhong Kang, Hang Ruan, Richard O. Claus, Jean Heremans, Marius Orlowski

**Affiliations:** Bradley Department of Electrical and Computer Engineering, Virginia Tech, Blacksburg, VA 24061 USA; NanoSonic Inc., 158 Wheatland Drive, Pembroke, VA 24136 USA; Physics Department, Virginia Tech, Blacksburg, VA 24061 USA

**Keywords:** Graphene nanoribbons, Partial and integer quantum conductance, Electron waveguide, Transmission coefficient, Memristor, Resistive memory

## Abstract

Quantized conductance is observed at zero magnetic field and room temperature in metal-insulator-metal structures with graphene submicron-sized nanoplatelets embedded in a 3-hexylthiophene (P3HT) polymer layer. In devices with medium concentration of graphene platelets, integer multiples of *G*_o_ = 2*e*^2^/*h* (=12.91 kΩ^−1^), and in some devices partially quantized including a series of with (*n*/7) × *G*_o_, steps are observed. Such an organic memory device exhibits reliable memory operation with an on/off ratio of more than 10. We attribute the quantized conductance to the existence of a 1-D electron waveguide along the conductive path. The partial quantized conductance results likely from imperfect transmission coefficient due to impedance mismatch of the first waveguide modes.

## Background

Graphene [1] has attracted much attention owing to its extraordinary properties such as quantum electronic transport [2, 3], a tunable band gap [4], extremely high-mobility [5], and high elasticity modulus [6]. One of the most promising characteristics of graphene is the ability of charge carriers to travel through it ballistically over hundreds of nanometers. Recent developments in the preparation of high-mobility graphene [7, 8] made it possible to study the effects of quantum confinement in graphene nanostructures. Tombros et al. [9] observed quantized conductance at integer multiples of *G*_o_ = 2*e*^2^/*h* (12.91 kΩ^−1^ = 77.48 μS; here, *e* is the elementary charge and *h* is Planck’s constant) in a suspended graphene nanoconstriction at cryogenic temperature of 4.2 K. The observed quantized conductance was attributed to the graphene zigzag or armchair edges that provide effective boundary conditions for the quantum mechanical wave functions. In 1998, Frank et al. [10, 11] observed conductance of multiwalled carbon nanotubes (sheets of graphene rolled up into cylinders) to be quantized at room temperature. The experiments were performed using multiwalled carbon nanotubes attached to the tip of a scanning probe microscope which is gradually lowered into a liquid mercury contact. The conductances measured in the experiment are consistent with 1*G*_o_ conductance per nanotube. Calculations by Sanvito et al. [12] have shown that inter-wall interactions between adjacent nanotubes not only block some of the quantum conductance channels but also redistribute the current non-uniformly over an individual tube and modify the density of states near the Fermi level, giving rise to integer and non-integer quantized conductance values. Observations of integer and fractional quantum Hall effects in graphene systems reported [13, 14] are not further discussed in the context of this paper, as they presuppose existence of Landau levels that can be ruled out in our case of absent magnetic field. Nevertheless, irrespective of the origin of quantized conductance, phenomena related to quantized conductance in graphene composites at ambient temperatures are desirable since they would provide added freedom, on the one hand, to investigate finer features of quantized conductance, and on the other, to develop graphene-based applications of quantum technologies, be it as devices or memory cells. The impact of graphene on memristive switching behavior has been investigated in graphene oxide layers [15–17] in tantalum oxide layers with engineered nanopores filled with graphene [18] showing large hysteresis I-V characteristics. Also, graphene quantum dots, embedded in a semiconductor polymeric matrix, act as charge trapping nanomaterials leading to I-V characteristics with a pinched hysteresis loop [19].

## Methods

Here, we present experimental observation of integer and partially quantized conductance at room temperature in metal-insulator-metal (MIM) structures consisting of graphene nanoplatelet (GNP) ribbons suspended in a 3-hexylthiophene polymer (P3HT) layer interposed between metal copper and gold electrodes.

### Device Fabrication

**Fig. 1:**
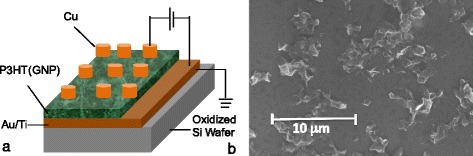
**a** Schematic of the graphene-based organic MIM structure; **b** SEM image of the pristine graphene nanoplatelets deposited on an oxidized silicon wafer substrate

Our graphene-based MIM devices have been fabricated on a thermally oxidized silicon wafer using standard semiconductor processes. The thermally oxidized silicon wafer was first cleaned using acetone and subsequently rinsed in isopropanol and deionized water. The wafer was then patterned photolithographically and deposited with a bottom gold (Au) electrode using electron-beam physical vapor deposition (EBPVD). GNP powder provided by PPG Industries [20] was first dispersed in toluene and further exfoliated for 1 hour by ultrasonication. P3HT was subsequently dissolved into the GNP solution and ultrasonicated for another hour. According to the manufacturer [21, 22], GNP comprises one or more layers of one-atom-thick planar sheets of sp^2^-bonded carbon atoms densely packed in a honeycomb crystal lattice. The number of stacked layers is typically between 5 and 30. The lateral dimension of the flakes ranges between 100 nm and a few micrometers. The graphene platelet ribbons are substantially flat, but when made thin by exfoliation, they can be curved, curled, or buckled indicating a single flake or a stack of a few carbon sheets [21, 22]. Ultrasonication has been found to be a very effective method in overcoming the van der Waals forces between the individual carbon sheets leading to uniform dispersions of single flakes or very thin stacks of flakes. The properties of the graphene nanoplatelets used are described in more detail in refs. [20–22]. The resulting P3HT(GNP) dispersion was then drop-deposited onto the bottom metal Au electrode and covered by islands of copper (Cu) electrodes also using EBPVD deposition to form Au/P3HT(GNP)/Cu devices. The thickness of the P3HT(GNP) film is 700 nm, and its uniformity is +/−50 nm. Spin coating of the dispersion has been tried but resulted in poor adhesion to the subjacent Au electrode. The graphene concentrations in the P3HT(GNP) solutions vary from 0.05 to 0.2 mg/ml. The P3HT solution is 10 ml. The devices have a square-shaped area of the Cu electrode of 2 mm × 2 mm and of 3 mm × 3 mm. The thicknesses of the layers are 60, 700, and 150 nm, for gold, P3HT(GNP), and copper, respectively. The P3HT(GNP) layer is thick enough to accommodate non-horizontally positioned GNP ribbons. For each concentration, ten devices with the same structure and the same active area were fabricated. For comparison and assessment of the role of carbon nanoplatelets, six Au/P3HT/Cu structures have been fabricated with no graphene nanoplatelets. All of these devices show no quantized conductance effects. The manufacturing process flow of the devices is (i) a 60-nm gold layer is evaporated by EBPVD on oxidized 4-in. wafers. The Au electrode is a common ground plate to all devices on the wafer, and (ii) the P3HT(GNP) solution is drop-deposited onto the surface of the Au electrode. (iii) The top islands of copper (Cu) electrodes are deposited by EBPVD on top of the P3HT(GNP) film using a shadow mask. The thickness of the three layers has been verified by SEM cross-sections. The schematic of the graphene-based MIM structure is shown in Fig. 1a. Figure 1b shows a scanning electron microscopy (SEM) image of GNPs dispersed first in an organic solvent, toluene, (not P3HT) and then after ultrasonification, dried the deposit by evaporating toluene on oxidized Si wafer to characterize the size and shape of the GNP platelets. Figure 1b is the top view of GNP flakes on the wafer. When graphene flakes are dispersed into P3HT, they cannot be seen in SEM images. Dispersion of graphitic nanoparticles in polymers is an active field of research aiming at the development of promising new materials [23–26]. We note that GNP powders from four manufacturers have been used, and only the devices with GNP powder from PPG Industries showed the quantized conductance effects. Our SEM images confirm that after ultrasonication, GNPs appear thinner and flake-like. It is, however, difficult to ascertain analytically whether the exfoliated GNPs consist of one or a few graphene layers. Nevertheless, the frequently observed warping points to the existence of very thin ribbon stacks consisting of very few and possibly one graphene layer. SEM cross-sections did not provide any further evidence as single layers and thin stacks of graphene could not be distinguished.

Four types of samples have been manufactured with regard to GNP content in the polymer: (i) no GNP, (ii) 0.05 mg/ml, (iii) 0.1 mg/ml, and (iv) 0.2 mg/ml of GNP concentration.

## Results and Discussion

### Electrical Characterization

**Fig. 2:**
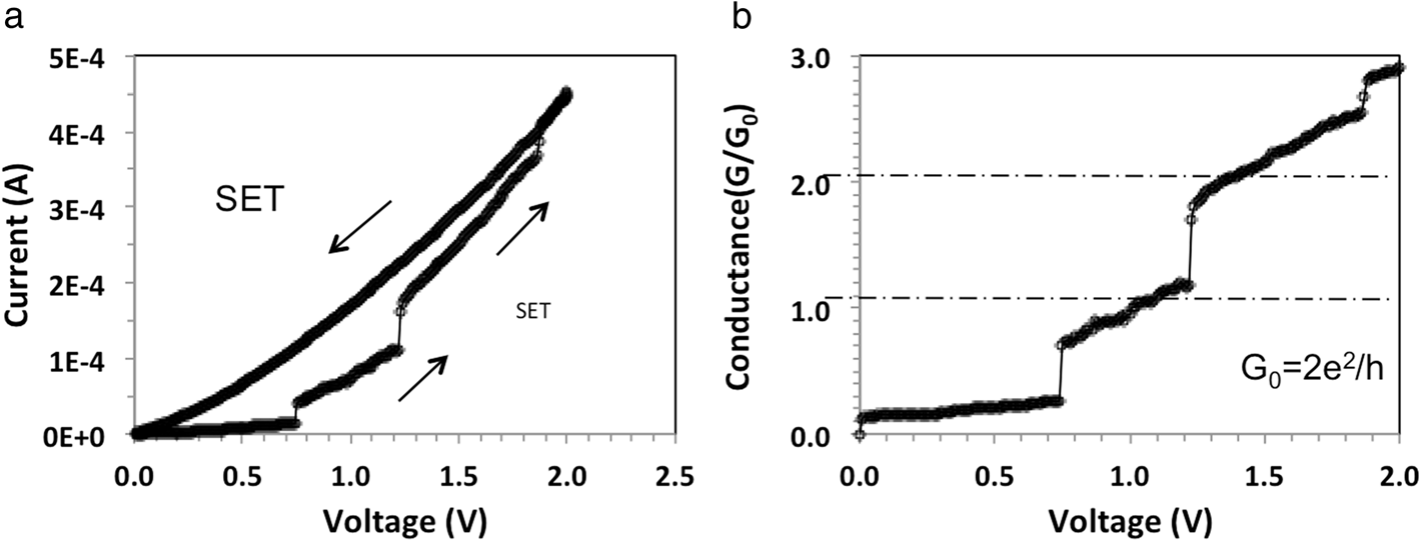
**a** I-V characteristics of an Au/GNP/P3HT/Cu device (GNP concentration: 0.1 mg/ml); **b** Conductance as function of voltage

**Fig. 3:**
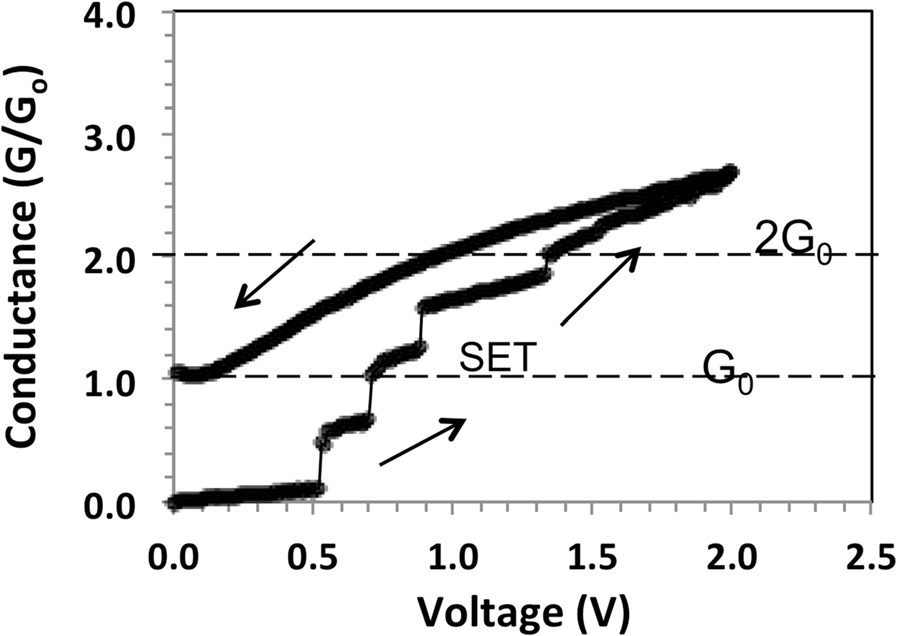
Conductance as function of voltage for the same device as in Fig. 2 for a subsequent data set

**Fig. 4:**
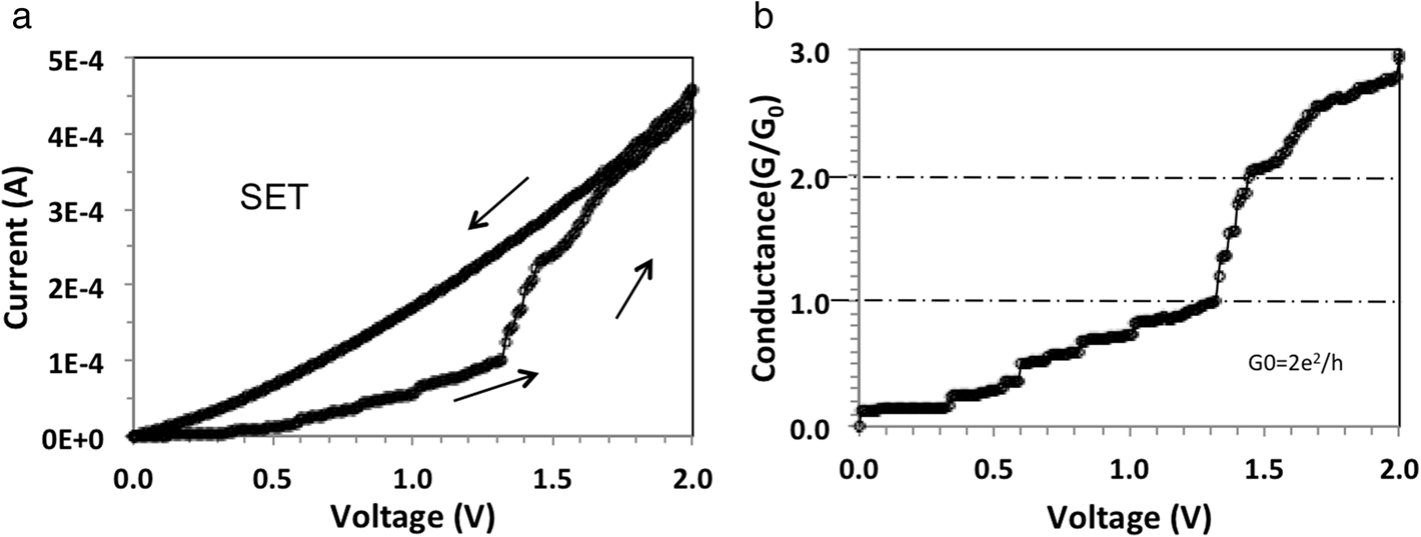
**a** I-V characteristics of an Au/GNP/P3HT/Cu device (GNP concentration: 0.1 mg/ml); **b** Conductance as function of voltage

**Fig. 5:**
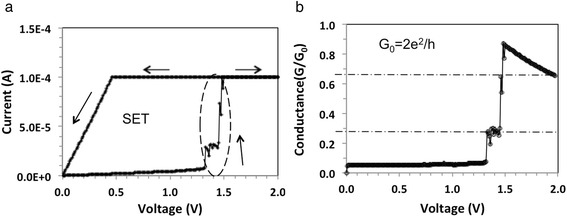
**a** I-V characteristics of an Au/GNP/P3HT/Cu device (GNP concentration: 0.1 mg/ml) with a *I*_cc_ = 0.1 mA; **b** Corresponding conductance as function of voltage extracted from Fig. 5a

**Fig. 6:**
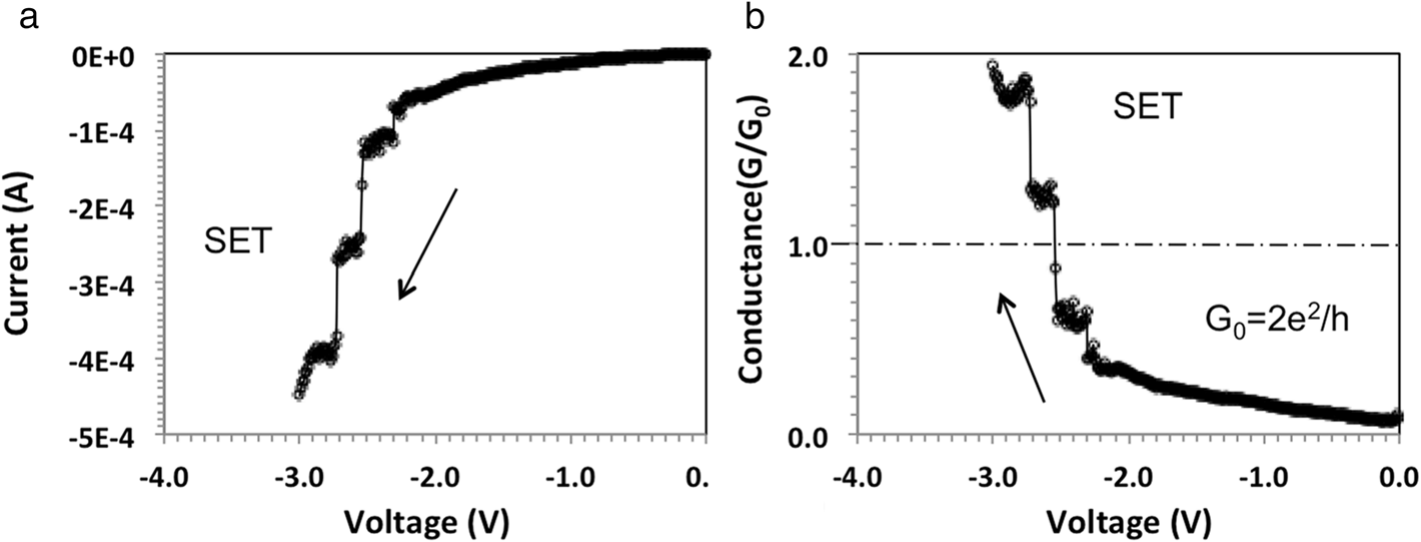
**a** I-V characteristics of the set operation of the same device as in Fig. 2, at negative voltage bias. **b** Corresponding conductance as function of voltage extracted from Fig. 6a

From these four types of devices, only devices with 0.1 mg/ml show integer and partial quantized conductance as shown in Figs. 2, 3, 4, 5, and 6. In general, roughly 25 % of the 0.1 mg/ml devices display integer quantized conductance, 25 % partial quantized conductance, and remaining 50 % no quantized conductance. By integer or partial quantized conductance, we mean that the two-contact conductance of the device shows transitions in integer units of *G*_o_ or repeatable fractions of *G*_o_, respectively, as function of applied voltage. Figure 2a shows typical current-voltage (I-V) characteristics. Starting at zero voltage, the voltage is first swept at a fixed ramp rate *υ* = 0.225 V/s (*V*(*t*) = *υ* × *t*) along the positive x-axis from 0 to 2 V. The current is observed to increase gradually and ohmically in the low-voltage region, and then abruptly increase at three voltages, specifically at 0.75, 1.2, and 1.87 V to 10, 162, and 409 μA, respectively. The initially high-resistance state (HRS) of about *R*_off_ = 100 kΩ is reduced during the voltage sweep to a final low-resistance state (LRS) of *R*_on_ = 5 kΩ. Figure 2b shows the conductance as a function of voltage for the same device. It can be clearly seen that the conductance during the transition from HRS to LRS is quantized roughly in multiples of *G*_o_. Conductance increases with voltage discretely, and instead of displaying flat plateaus as in quantum Hall effects, it displays sloped rises whose slopes decrease with the subsequent two steps. To characterize this behavior in terms of energy consumed, we have calculated the energy consumed between individual discrete jumps. For the first slope we obtain, assuming a linear voltage ramp rate *υ*:

$$ {E}_{\mathrm{step}}(1)={\displaystyle \underset{t_1}{\overset{t_2}{\int }}I(t)\cdot V(t)dt={\displaystyle \underset{V_1}{\overset{V_2}{\int }}\left({G}_0/\nu \right)\cdot {V}^2dV=\frac{G_0}{3\nu }}}\left({V}_2^3-{V}_1^3\right) $$

Using the data from Fig. 2b for first step (*V*_1_ = *V*(*t*_1_) = 0.72 V, *V*_2_ = *V*(*t*_2_) = 1.22 V, and *υ* = 0.225 V/s) we obtain *E*_step_(1) = 1.03 × 10^15^ eV. As this number is many orders of magnitude higher than kT, thermal effects can be ruled out for being responsible for the sloped conductance. Assuming that somewhere in the device a one-dimensional filament is being formed, one could attribute the slope to progressive thickening of the conductive filament constituting a waveguide. Another possible explanation is that the transmission coefficient of the electron waveguide is a function of the applied voltage. When the transmission coefficient is close to unity, it causes an integer quantized conductance jump. In Fig. 3, a subsequent set operation is shown for the same device after it has been reset to HRS state. The voltage is ramped again from 0 to 2 V and then back to 0 V. The new set I-V characteristic displays also quantized conductance steps, but in this case, the jump height appears to be 1/2*G*_o_ instead of n*G*_o_ as in Fig. 2b. Figures 2 and 3 show typical behavior of our devices where the I-V characteristics are seldom reproducible exactly but always display similar pattern of discrete steps for its conductance. Figure 4 shows the I-V characteristics of a different device but with same GNP content of 0.1 mg/ml, where in this case, integer quantized conductance and partial quantized conductance jumps coexist. Upon a voltage sweep from 0 to 2 V and then from 2 back to 0 V, Fig. 4a shows similar hysteresis as the I-V characteristic in Fig. 2a. Figure 4b shows the data in terms of conductance, extracted from the I-V characteristic in Fig. 4a. In the region *V* = 1.3 V to *V* = 1.45 V, it can be seen that the conductance changes abruptly from 1*G*_o_ to 2*G*_o_ and above *V* = 1.5 V rises from 2*G*_o_ to 3*G*_o_. In addition, from *V* = 0.0 V to *V* = 1.3 V, a sequence of smaller conductance steps within the 0*G*_o_ to 1*G*_o_ interval is also observed, indicating that the partial quantized conductance coexists with integer quantized conductance. The best fit to the partial quantized conductance within the 0*G*_o_ to 1*G*_o_ interval can be obtained by assuming a fraction v*G*_o_ with *v* = 1/7, 2/7, 3/7, 4/7, 5/7, and 6/7, although 3/7 is slightly off the experimental value. The resolution of the partial conductance steps between 1*G*_o_ and 2*G*_o_ is always much less pronounced and appears to follow the same multiples of 1/7. Steps with 8/7, 9/7, 11/7, and 13/7 can be clearly identified. In the third conductance step, from 2*G*_o_ to 3*G*_o_, only a rudimentary partial quantized conductance can be discerned. The behavior shown in Fig. 4b has been observed for several devices and can be repeated on the same device with a slightly changed pattern and length of the steps. For memory applications, the I-V hysteresis shown in Fig. 2a and in Fig. 4a can be made more pronounced when a compliance current *I*_cc_ is imposed during the set operation. Such a case is shown in Fig. 5a with *I*_cc_ = 0.1 mA. It can be seen that the hysteresis is now pronounced and readily detectable as a difference in logic state “0” for off-state and “1” for on-state. The conductance observed during the set transition of 1.4 V has been extracted and is plotted in Fig. 5b showing partially quantized conductance. Finally, we address the reproducibility of the electric characteristics. The I-V characteristics shown in Figs. 2a, 4a, and 5a could be reproduced qualitatively not more than 10–14 times. The characteristics were never identical even for the same device, but the quantum conductance steps have always been observed in the same voltage interval between 0.5 and 1.4 V. However, it should be kept in mind that these devices demonstrate a general principle and underlying mechanisms rather than function as devices optimized for a particular application.

### Memristive Properties of the Devices

**Fig. 7:**
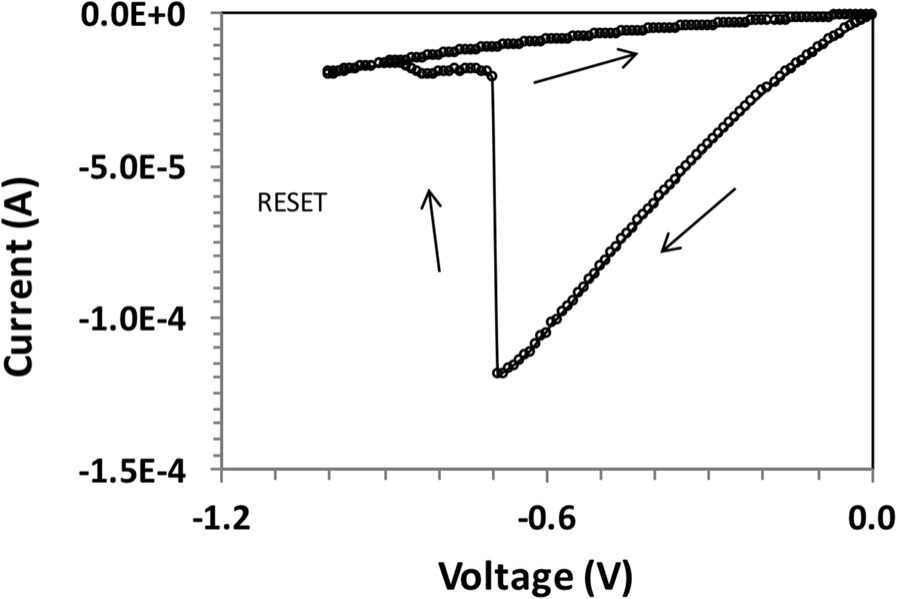
A reset operation; a sharp transition at *V*_reset_ = −0.7 V can be observed from LRS to HRS when the current drops abruptly by two orders of magnitude

As we consider Au/P3HT/Cu cells for potential memory applications, we have performed the set operation under imposition of the so-called compliance current (*I*_cc_), which is commonly used in resistive random access memory devices less the device be damaged. *I*_cc_ is the maximum current that is allowed to flow through the device. In resistive random access memory, devices under the *I*_cc_ limitation quantized conductance is also observed when a fresh device is subjected to negative bias. In Fig. 6a, the I-V characteristics for a “set” operation at a negative bias is shown. Figure 6b shows the extracted conductance as a function of voltage. Again, partial conductance steps can be observed. Interestingly, quantized conductance has never been observed during the “reset” or erase operation. A typical reset operation of the device is shown in Fig. 7. A sharp reduction by almost two orders of magnitude at *V*_reset_ = −0.7 V can be observed.

### Explanation of the Observed Quantum Effects

There are several reports on integer and partial quantized conductance observed in resistive switching devices [27–29]. Integer quantized conductance has been observed in resistive switching devices at 300 K and attributed to the formation of Cu nanofilament in TaO_x_ between the metal electrodes [27]. Li [28] and Csonka [29] have reported partial quantized conductance measured on Cu and Au nanowires, respectively. They attributed the partial, non-integer values as expressed in terms of *G*_o_ to the presence of adsorbed organic molecules and hydrogen, respectively, adsorbates which disturb the conductance through the nanowires.

**Fig. 8:**
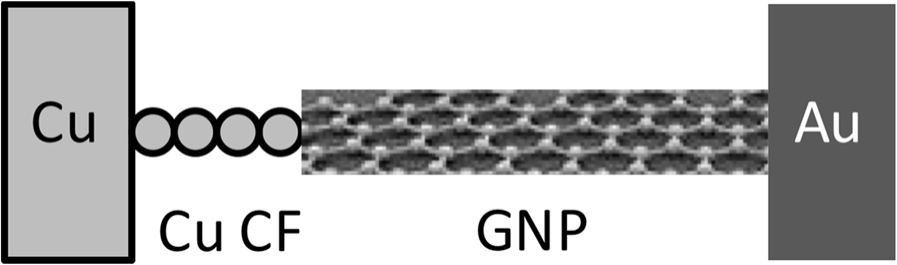
Formation of 1-D waveguide between Cu electrode and graphene nanoplatelet (GNP) in terms of a few Cu atoms

Following the explanation adopted for Cu and Au nanofilaments and quantum point contacts in mesoscopic physics [30, 31], we explain the observed quantized conduction in terms of a 1-D electron waveguide. Since our explanation relies on at least on a partial formation of a Cu or Au filament which would make the bulk of the observed on-resistance of ca 5 kΩ, we have performed temperature coefficient of resistance (TCR) measurements of the R_on_ state between 300 and 355 K. The TCR that we measured is 0.0029 K^−1^ which is close to the TCR = 0.0033 K^−1^ of a Cu filament in Cu/TaO_x_/Pt devices [32, 33]. The value of TCR = 0.0033 K^−1^ has been measured for *R*_on_ = 310 Ω. We have found that for higher resistance values *R*_on_ of the filament, the TCR decreases. Hence, TCR = 0.0029 K^−1^ for *R*_on_ = 5 kΩ is consistent with values found for Cu filaments in Cu/TaO_x_/Pt devices at higher *R*_on_ values. The attribution of the resistance to the Cu filament and not to a possible Au filament is also consistent with the much lower ionization of Cu → Cu^+^ + e^−^ (7.73 eV) than the ionization energy for Au (9.23 eV).” As shown in Fig. 8, the waveguide is effectively realized by a 1-D conducting bridge consisting of a few Cu atoms between one of the electrodes and a graphene nanoplatelet or between two graphene nanoplatelets, dominating the resistance of the device. Figure 8 shows the case of a 1-D conducting bridge formed by a few of Cu atoms between a graphene platelet and Cu electrode. The other possibility of two graphene platelets in contact with the electrodes and the Cu atoms providing a 1-D bridge between the two graphene platelets is rather speculative, as the Cu migration along the GNP ribbons appears to be quite unlikely. The ionized Cu^+^ ions migrate across the polymer and reach a GNP platelet where they are reduced to Cu atoms. Under a positive bias applied to the Cu electrode, Cu^+^ ions may be injected into the polymer and form a conductive filament between the Cu electrode and graphene platelets whose other end makes a contact with the Au electrode. As a result of this, the Cu^+^ ions could aggregate more densely in the polymer than along the graphene surfaces and form tiny nanofilaments connecting the graphene layers to each other or to the electrodes. One observes that when a negative bias is applied to the Cu electrode, a strong enough field is able to ionize the neutralized Cu atoms of the nanofilaments and rupture them, disconnecting them from the graphene nanoplatelets.

**Fig. 9:**
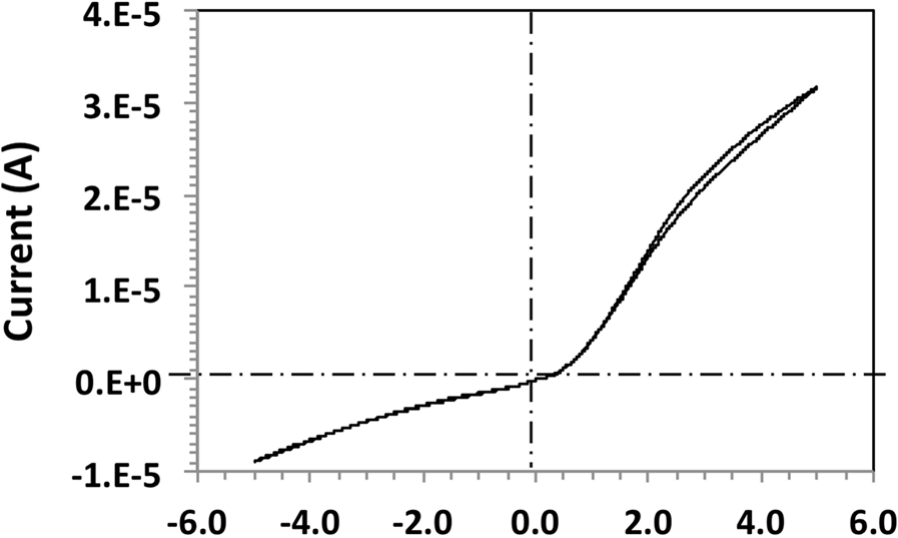
I-V characteristics of an undoped P3HT polymer layer (no graphene nanoplatelets) with currents significantly lower than for doped polymer with GNP concentration larger than 0.05 mg/ml

The 1-D conducting bridge forms a constriction of width of the order of the Fermi wavelength of the electron. For a width approximately half of the Fermi wavelength, one transverse mode will contribute to conductance (1*G*_o_), for wider constrictions, second and higher modes are transmitted. Each mode contributes a unit *G*_o_ to the conductance if the transmission coefficient of the mode through the constriction is unity (perfect transmission without backscattering) [30, 31]. Yet, the transmission coefficients of the modes depend on the environment of how the constriction connects (the equivalent of an electromagnetic impedance mismatch) to the graphene platelets and the two metal electrodes. A non-unity transmission coefficient results in a correspondingly reduced and partial quantized conductance. The addition of partially transmitted modes then results in the observed partial quantized conductance. The observed slope of conductance vs voltage follows from the observation that the transmission coefficients of the modes through the electron waveguide are a function of the applied voltage. This is a reasonable assumption given the observed imperfect transmission and the fact that the applied voltage will change the electrostatic environment of the constriction, thereby changing the transmission coefficients as well. The role of graphene platelets, apart from providing a conductive path, is to provide a 2D electron system with a Fermi wavelength that is readily tunable by a change in the electrostatic environment and hence by the applied voltage. This explains why we see the quantum effects only at medium concentration of graphene platelets. At medium concentration, a high chance exists to form a single 1-D electron waveguide somewhere in the device, dominating the resistance and with a constriction size of the order of the Fermi wavelength that can be modulated by the applied voltage. At high concentration of graphene platelets, many individual graphene platelets or stacks of graphene platelets form a multitude of parallel conductive paths and the quantum effects are averaged out. At low concentrations, the probability of forming even one bridging constriction is low. It should be noted that the area cross section of our devices is large (<0.09 cm^2^). At a GNP concentration of 0.1 mg/ml, we find a 50 % chance that one graphene nanoribbon will span the entire dielectric distance. At a GNP concentration of 0.05 mg/ml, not a single case of bridging has been observed. On the other hand, at concentration of 0.2 mg/ml, the very high measured conductance value points to multiple graphene nanoribbons in parallel bridging the two metal electrodes. While the highly conductive devices with GNP concentration of 0.2 mg/ml do not display quantized conductance effects, they are a good material candidate for stretchable transparent electrodes [34]. On the other hand, undoped P3HT polymer behaves as a insulator as shown in the I-V characteristics of Fig. 9, with almost no hysteresis effect. The broad range of electric tunability of organic films points to the universal potential of graphene nanoplatelets, depending on their concentration in an organic dispersion, for a wide spectrum of applications.

The non-unity transmission coefficients are caused by the sensitivity to the electrostatic environment. Likewise, in memristive devices, in contrast to conventional resistors and capacitors, the resistor and capacitor [35] are voltage or current dependent, or more generally, they depend on the total voltage flux (defined as the time integral of the applied voltage) or on the total charge (defined as the time integral of the applied current). Accordingly, we would have an experimental demonstration of a quantum memristor, achieved by the sensitivity to the electrostatic environment, of the transmission coefficients through an electron waveguide.

## Conclusions

In summary, we have observed integer and partial quantized conductance occurring in graphene nanoplatelets doped organic polymer films at room temperature. The quantized conductance is likely a result of the formation of a 1-D waveguide with a voltage-tunable transmission coefficient. The device shows a distinct I-V hysteresis and lends itself for memory applications^1^. The control of concentration of graphene nanoplatelets in flexible polymer layers allows the tuning of the material from insulator via quantum conductor to a metallic conductor, opening thus new vistas in electronic applications at low energy and at a low manufacturing cost. The present device is a realization of quantized conductance resistor with memory (memristor) in which the physical conduction is governed by integer and partial quantized conductance effects.

## Abbreviations

EBPVD: electron-beam physical vapor deposition
GNP: graphene nanoplatelet
HRS: high-resistance state
LRS: low-resistance state
MIM: metal-insulator-metal
P3HT: 3-hexylthiophene polymer
ReRAM: resistive read access memory
SEM: scanning electron microscopy

## Footnotes

1 The authors believe that the device demonstrated is the first reported realization of a quantum memristor.
